# Comprehensive evaluation of candidate reference genes for qRT-PCR studies of gene expression in mustard aphid, *Lipaphis erysimi* (Kalt)

**DOI:** 10.1038/srep25883

**Published:** 2016-05-11

**Authors:** Murali Krishna Koramutla, Raghavendra Aminedi, Ramcharan Bhattacharya

**Affiliations:** 1 ICAR-National Research Centre on Plant Biotechnology, Indian Agricultural Research Institute Campus, New Delhi-110012, India

## Abstract

Mustard aphid, also known as turnip aphid (*Lipaphis erysimi*) is a major insect pest of rapeseed-mustard group of crops. Tremendous economic significance has led to substantial basic research involving gene-expression studies in this insect species. In qRT-PCR analysis of gene-expression, normalization of data against RNA variation by using appropriate reference gene is fundamental. However, appropriate reference genes are not known in case of *L. erysimi*. We evaluated 11 candidate reference genes for their expression stability in 21 samples of *L. erysimi* subjected to various regimes of experimental treatments. Unlike other studies, we validated true effects of the treatments on the samples either by gene-expression study of an associated marker gene or by biochemical tests. In the validated samples, expression stability of the reference genes was analysed by employing four different statistical softwares geNorm, NormFinder, BestKeeper and deltaCt. Drawing consensus on the results from different softwares, we recommend three best reference genes *16S, RPS18* and *RPL13* for normalization of qRT-PCR data in *L. erysimi*. This study provides for the first time a comprehensive list of suitable reference genes for mustard aphid and demonstrates the advantage of using more than one reference gene in combination for certain experimental conditions.

Mustard aphid, also known as turnip aphid *(Lipaphis erysimi* Kaltenbach), inflicts heavy damage in several oilseed and vegetable Brassicas^1^. Particularly in rapeseed-mustard it has evolved as the most dreaded insect-pest in tropical and subtropical regions including India^1^,^2^. Genetic resistance against aphids is either unavailable or limited to a few wild accessions of *Brassica* coenospecies. Therefore, extensive basic research on physiological aspects of aphid biology with plausible implication in its integrated management strategy is being perused worldwide^3^,^4^. In this context, gene-expression studies of important aphid genes, involved in parasitic lifecycle, have assumed significance in entomological research. An immediate application of such studies, for example, has led to several attempts for developing RNAi mediated host-resistance by targeted silencing of aphid genes^5^,^6^,^7^,^8^,^9^.

qRT-PCR has rapidly gained importance as a robust method for studying gene-expression because of its high sensitivity and convenience in large throughput. However, certain limitations such as, batch to batch variation in output, variable efficiency of reverse transcription and PCR reaction which influences the threshold (Ct) values have rendered this technique skill-intensive^10^. Consequently, the technical challenges of attaining precision in qRT-PCR based analysis depend on appropriate selection of the reference gene(s) in the experimental set up. Commonly, housekeeping genes are used as the reference genes with the fundamental assumption that their expressions remain constitutive and unaffected irrespective of the treatments or changes in physiological condition of the samples during the course of the experiments^11^,^12^,^13^. Accordingly, any sample to sample variation indicated by change in expression data of the reference gene is considered as art-effect and the same is nullified in the output data through the process of ‘normalization’. Therefore, much credibility of the qRT-PCR data relies on true constitutive expression of the reference gene across the samples under study.

For qRT-PCR based gene-expression studies in insects including a few aphid species, housekeeping genes such as *18S rRNA, ACT1, EF-1a, GAPDH, UBI, RPSs, RPLs* and *TUB* have been commonly used as reference genes^6^,^12^,^14^,^15^,^16^,^17^,^18^. However, several recent reports on expression inconsistency of these genes question their suitability as reference genes in qRT-PCR studies (reviewed by Gutierrez *et al*.^19^; Kozera and Rapacz^20^). In fact, it has now been shown that most of the house keeping genes demonstrate variable expressions depending on the organism, its developmental stage and also the treatment conditions^21^,^22^,^23^,^24^. Therefore, none of these genes can be universally used as reference gene; rather a validation of their suitability as reference genes in the target species is a prerequisite.

Owing to the importance of aphids as the most significant insect-pest of rapeseed-mustard there have been an increasing number of scientific reports on functional genomics of its biology as well as annotated gene sequences^25^,^26^. In most of the aphid species including *L. erysimi*, qRT-PCR studies are carried out with non-validated reference genes commonly based on their use in other organisms. Report on validation of references genes for qRT-PCR, though rapidly being available in other organisms, is lacking for this aphid species. As a result, the impulse for appropriate reference gene for *L. erysimi* system is felt in many of the research studies that involve qRT-PCR. In this context, the current study was undertaken for validating 11 housekeeping genes, such as, *16S ribosomal RNA* (*16S*), *succinate dehydrogenase B* (*SDHB*)^27^, *actin* (*ACT*)^28^, *elongation factor 1a* (*EF1A*)^12^,^16^, *ribosomal protein L13a* (*RPL13*)^29^, *ribosomal protein S18* (*RPS18*)^15^, *ribosomal protein L27* (*RPL27*)^28^, *ribosomal protein L29* (*RPL29*)^12^, *β-tubulin* (*TUB*)^30^, *glyceraldehydes 3-phosphate dehydrogenase* (*GAPDH*)^18^, *arginine kinase* (*AK*)^30^, as reference gene for qRT-PCR in *L. erysimi.* The expression stability of these genes across the regime of different experimental conditions was evaluated by using four statistical algorithms, geNorm^11^, NormFinder^31^, BestKeeper^32^ and deltaCt method^33^. To the best of our knowledge it is the first report on comprehensive evaluation of the reference genes in *L. erysimi*. The outcome of the study will not only benefit those experiments which involve gene-expression study in this aphid species but will immediately find translational application in other closely related aphids.

## Results

### PCR amplification of candidate reference genes

**Figure 1:**
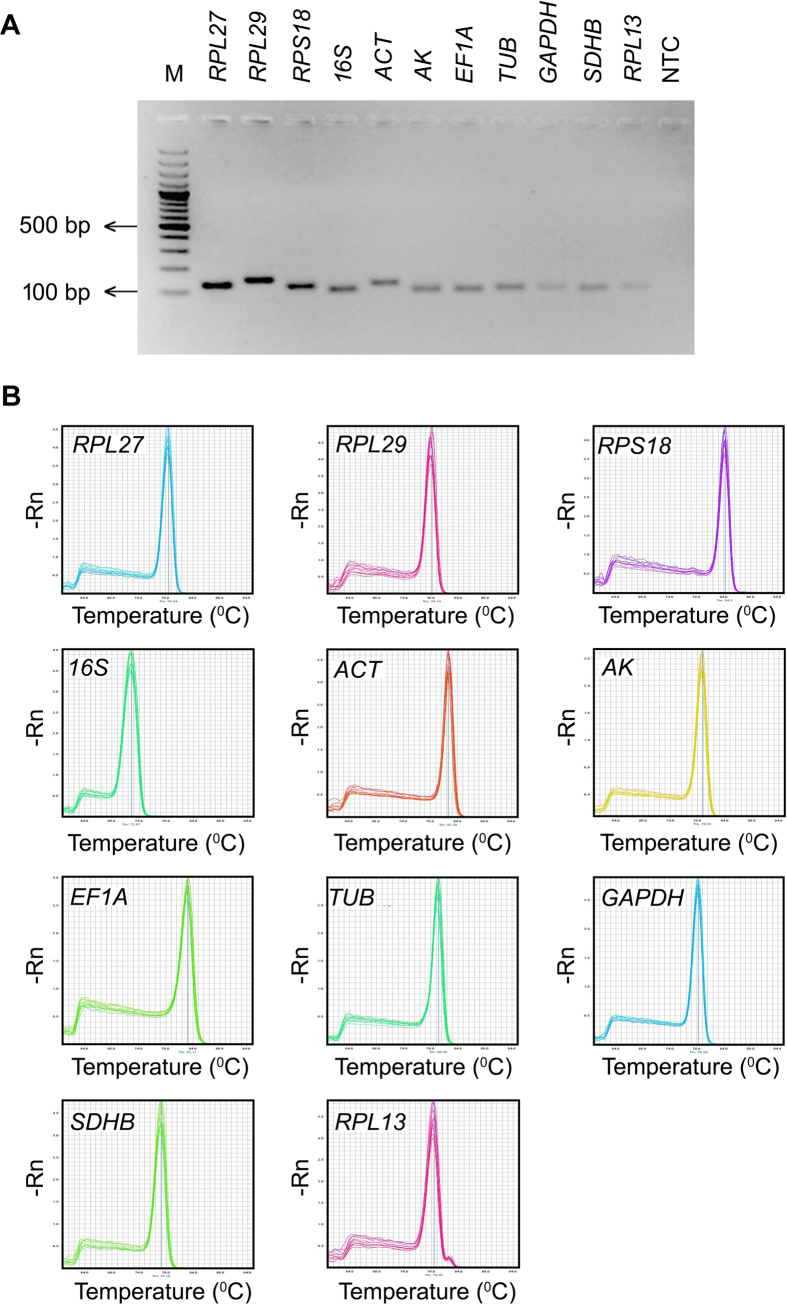
Amplification specificity of primers in RT-PCR and qRT-PCR. (**A**) Single amplicon of desired size for each gene and no amplification in no template control (NTC) visualized on 2% agarose gel; (**B**) Single peak in melt curve analysis.

The sequence information of the reference genes in *L. erysimi* was not available in the genbank database. Therefore, the primers were initially designed based on the *A. pisum* sequences (Table S1). The specificity of PCR amplification on *L. erysimi* cDNA was validated by single amplification of expected size in each case. The amplicons were sequenced and the sequence data were validated by homology to their heterologous counterparts (Table S2). Based on *L. erysimi* specific sequences of the reference genes, the qRT-PCR primers were designed. The specificity of primer binding in qRT-PCR was further confirmed by the desired amplicon, visualized on agarose gel and melt curve analysis (Fig. 1). Linear regression coefficient R^2^ of all the primer-pairs were ranged between 0.987–1.000, and the PCR efficiency determined by the standard curve ranged from 91.2–102.4% (Table S3; Fig. S1).

### Validation of treatment-effects by gene-expression and biochemical test

**Figure 2:**
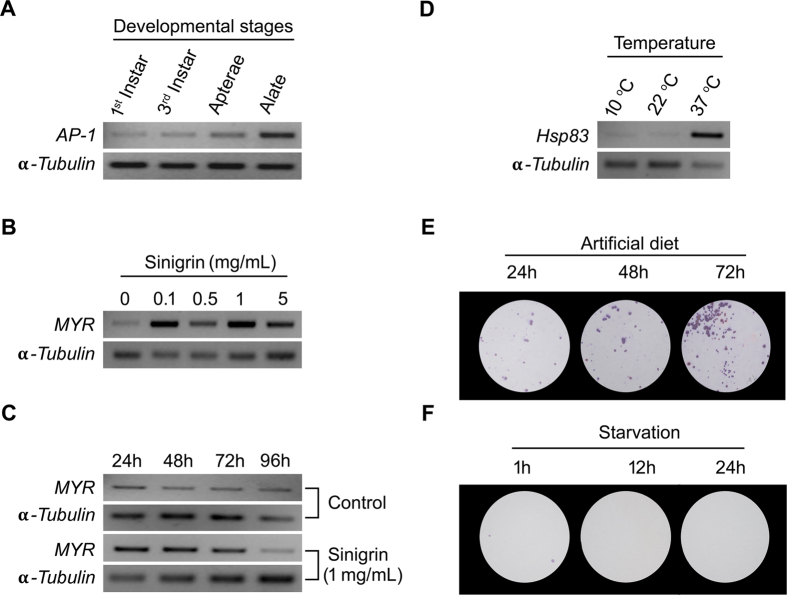
Validation of treatments by molecular markers and biochemical tests. (**A**) *AP-1* expression in four developmental stages; (**B,C**) Transcriptional activation of *MYR* in aphids due to sinigrin ingestion at different doses and for different duration; (**D**) Transcriptional activation of *Hsp83* by temperature stress; (**E**) Ninhydrin assay of honeydew secreted by aphids on artificial diet; (**F**) Ninhydrin assay for honeydews in starved aphids.

Treatment-effects on the *L. erysimi* samples were validated by assaying expression level of the marker genes in each case. In the samples of different developmental stages, a gradual increase in *AP-1* transcript level confirmed the stage-difference among the samples (Fig. 2A). For imposing antibiosis, the aphids were fed on diet supplemented with variable levels of sinigrin, and for variable duration. Ingestion of sinigirin led to enhanced expression of the *myrosinase* gene in the treated aphids (Fig. 2B,C). Similarly, the effect of temperature stress was verified by multifold induction of *Hsp83* transcripts at 37 °C compared to lower (10 °C) and optimal (22 °C) temperatures (Fig. 2D). Feeding of the aphids on artificial diet was validated by honeydew deposition detected through ninhydrin staining. A gradual increase in staining intensity was observed on the whatman paper with increasing feeding duration (Fig. 2E). As expected, in case of starvation no stain was detected (Fig. 2F).

### Expression profile of the candidate reference genes in *L. erysimi*

**Figure 3:**
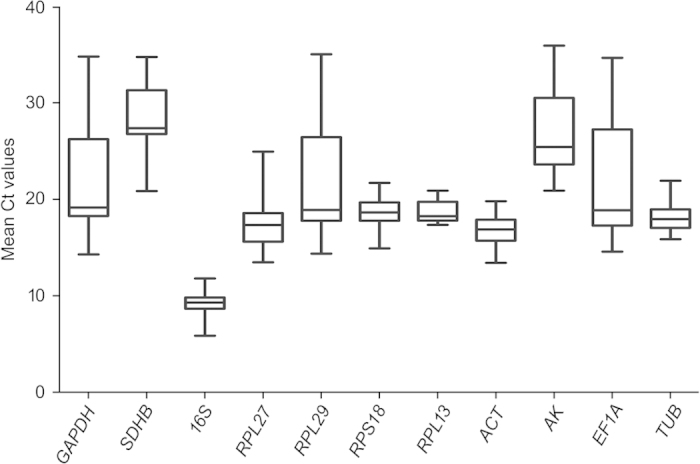
Whisker box plots of reference gene expression in 5 treatments. The boxes represent the mean Ct values and the line in the box represents median. The bars across the boxes show the minimum and maximum Ct values. Whiskers represent 95% confidence intervals. Data represent mean values ± SD (*3n* = 9).

In qRT-PCR, a variable Ct value of all the reference genes across the 5 treatments indicated that their gene-expression levels are affected by the treatments albeit at differential extents (Fig. 3). The expression of *SDHB, RPL29*, and *AK* were maximally affected by the developmental stages and temperature stress. Similarly, the expression of *EF1A, GAPDH, AK*, and *SDHB* varied significantly among the samples collected from aphids either fed on artificial diet for different durations or treated with sinigrin in the diet. In a comparative assessment, a narrow range of variable Ct values in case of *16S, RPS18* and *RPL13* across the type and time course of the treatments empirically suggested their relatively more stability of expression. In contrary, *RPL29, EF1A* and *GAPDH* displayed highest variations in Ct values when all the experimental treatments were taken into account. Irrespective of the treatments, *16S rRNA* was found to be most abundant, with the lowest mean Ct value 9.23, compared to the transcript levels of other protein coding genes (Fig. 3).

### Expression stability and ranking of the candidate reference genes

The candidate reference genes were ranked based on their gene-expression stability over the treatments by employing four statistical algorithms. In analysis by different softwares, the rank order of the reference genes for individual treatment showed similar trends with subtle variation, which may be attributed to the differences in algorithms. For the purpose of more clarity in depiction the ranking of the reference genes generated by each statistical method has been dealt separately.

#### geNorm

**Figure 4:**
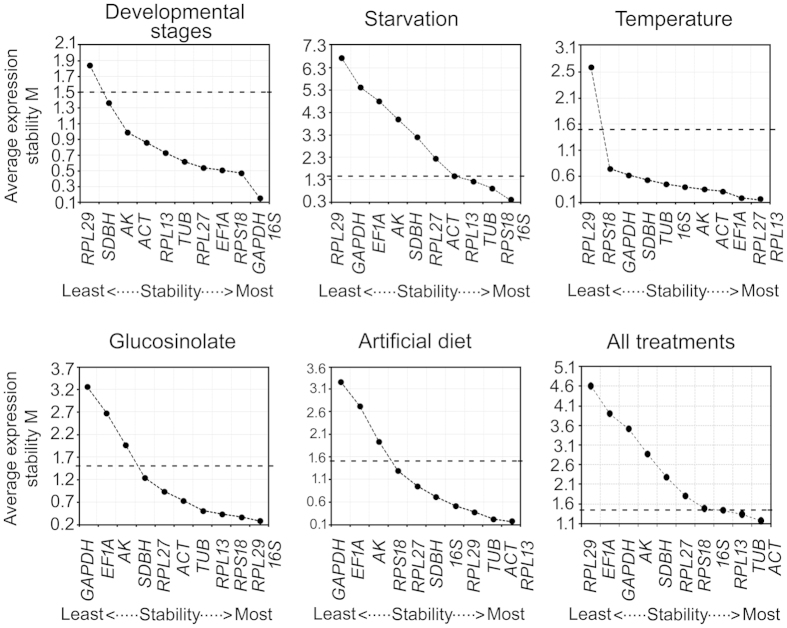
Expression stability and relative ranking of the 11 reference genes predicted by the geNorm. The mean expression stability (M) was calculated by stepwise exclusion of the least stable gene across all the samples within a particular group set. The mean stability of different genes is plotted; the least stable genes are represented on the left and the most stable on the right side of the plot. The dotted line indicates the threshold value of mean expression stability. The genes with M ≤ 1.5 are considered significant with stable expression.

**Figure 5:**
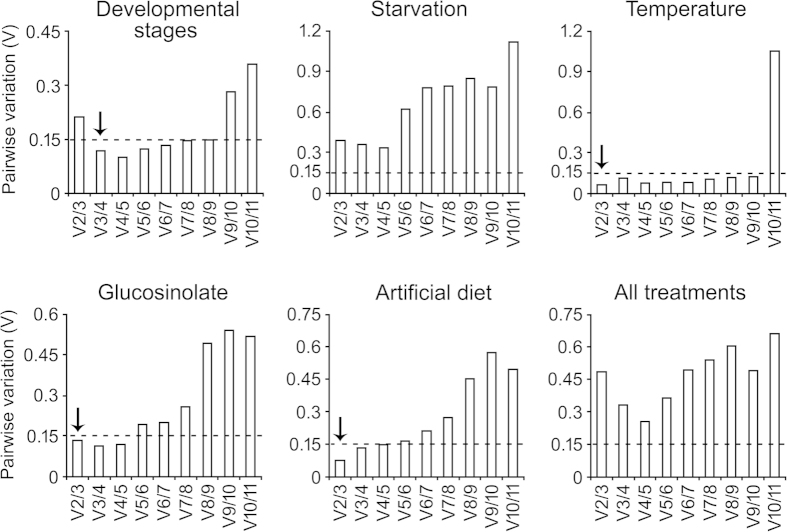
Optimal number of reference genes for accurate normalization calculated by geNorm analysis. Average pairwise variations (V) were calculated between the normalization factors NF_n_ and NF_n+1_ by geNorm software to indicate the optimum number of reference genes required for qRT-PCR data normalization. A threshold value of V ≤ 0.15 was suggested for significant normalization, the value below 0.15 indicated that the additional reference gene has no significant improvement on normalization in qRT-PCR data. *Arrow heads* indicate the optimal number of genes for normalization for that particular condition.

In geNorm analysis, all the reference genes significantly varied in their expression stability across the treatment conditions. More importantly, geNorm ranking unambiguously showed *16S* as the most stable reference gene across the treatments of developmental stages, starvation and antibiosis whereas *RPL13* was the best for the treatments of temperature stress and artificial diet (Fig. 4). However, taking into account all the treatments together, expression stability of *ACT* and *TUB* also scored below the threshold value along with *RPL13* and *16S*. Further, the optimal number of reference genes required for normalization of samples involving a specific treatment was calculated. If the pairwise variation (Vn/Vn + 1) between sequential normalization factors, NFn and NFn + 1 is >0.15, it is not necessary to use ≥n + 1 as an internal control for particular experimental conditions^11^. The pairwise variation V2/3 value was less than 0.15 for most of the conditions except in the case of developmental stages and starvation (Fig. 5). This indicated that combined use of two stable reference genes would be mandatory for normalization purpose in studies involving variable regimes of temperature, glucosinolate and artificial diet conditions. In developmental stages, the V3/4 value was less than 0.15, suggesting that the optimal number of reference genes for normalization would be three, namely *16S, GAPDH* and *RPS18*. However, in the case of starvation and all the treatments together, the pairwise variation values were above the cut-off value. The geNorm manual suggests use of minimum three most stable reference genes in such situations (Fig. 5).

#### BestKeeper

BestKeeper software tool calculates the standard deviation (SD) which is inversely proportional to the stability of expression. The rank order of the reference genes across the treatments suggested that no single reference gene can be used ideally for all the treatments (Table S4). The reference gene *16S* was most stably expressed and ranked the best in both starvation and temperature-stress conditions followed by *RPS18* and *RPL13*, respectively. Interestingly, gene-expression of *RPL13* and *RPS18* was least affected in antibiosis treatment compared to other treatments and ranked ahead of *16S*. In developmental stages, *GAPDH* showed more stable expression compared to *16S* and ranked as the best reference gene. Analysis of the aphid samples collected from the field validated similar trend in which *SDBH* and *GAPDH* were ranked as the best reference genes followed by *16S*. When the aphids were fed on artificial diet, expression of *RPL27* followed by *RPL29* was least affected among all the reference genes. In contrary, *RPL29* expression showed significant variation, in fact highest SD value, in samples of different developmental stages, starvation, and temperature stress.

#### NormFinder

Based on the rank order assigned by NormFinder algorithm either *EF1A* or *RPS18* were found to be most consistent in expression-level within and across the treatments, except starvation (Table S5). Under starvation *16S* was more stable and ranked higher than *RPS18*.

#### DeltaCt method

Delta Ct method identified *16S* as the most stable reference genes across the treatments of starvation, glucosinolate and temperature stress (Table S6). In developmental stage and artificial diet conditions *EF1A* and *ACT* were ranked as most stable, respectively.

### Validation of the reference genes

**Figure 6:**
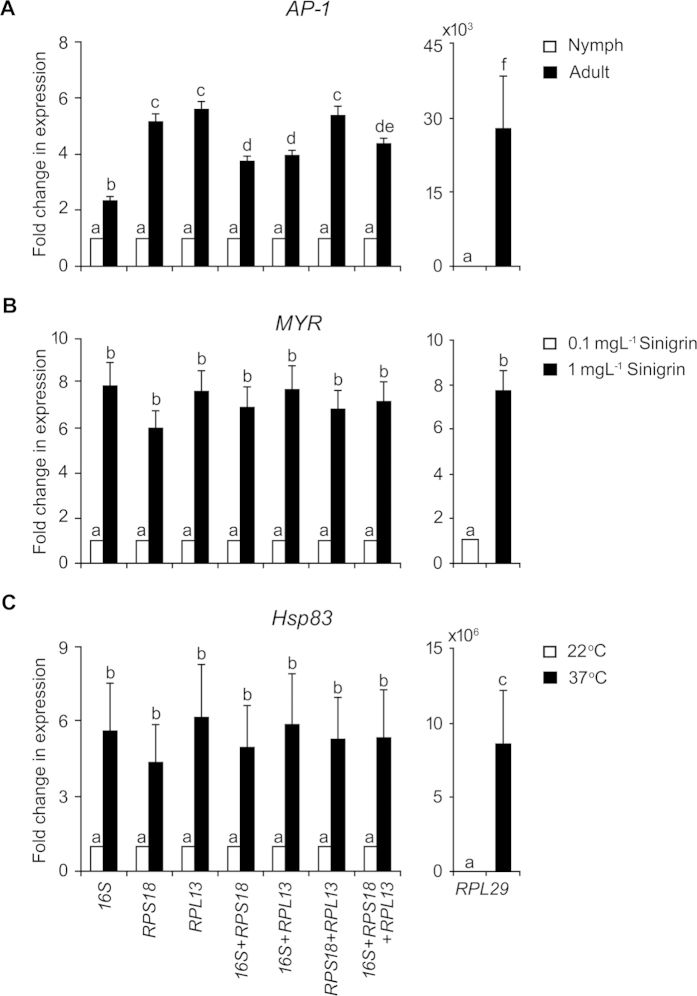
Variation in *AP-1, MYR* and *Hsp83* gene-expression data normalized by different reference genes and their combinations. Fold change in *AP-1, MYR* and *Hsp83* transcripts in aphids subjected to treatments of developmental stage (**A**), sinigrin (**B**) and temperature stress (**C**), respectively. Stable reference genes *16S, RPS18, RPL13,* their combinations and least stable reference gene *RPL29* were independently used as normalizer. Values represent mean ± SE (3*n* = 9) and comparison of means was carried out by student’s *t* test (*P* < 0.05). *Different letters* indicate significantly different values.

It was apparent that the best ranked reference gene for one treatment was not applicable to the other treatments. Therefore, for experimental convenience it was imperative to identify the reference genes that show acceptable, if not the highest, stability in expression within and across the treatments. Analysis of consolidated data indicated *16S, RPS18*, and *RPL13* as the top ranked reference genes in terms of stability in gene-expression for most of the treatment conditions. In contrary, *RPL29* was found to be the least stable. Applicability of these reference genes in normalization, either as single or in combination, was tested. This was carried out by assessing the transcript level of *AP-1, MYR* and *Hsp83* in the treatments of developmental stage, sinigrin and heat stress, respectively (Fig. 6). In case of developmental stage, either of *RPS18* and *RPL13* or combination of them as normalizer showed higher consistency in qRT-PCR data compared to normalization by *16S* and its combinations (Fig. 6A). However, for sinigrin treatment and heat stress normalization by a single recommended gene produced consistent and comparable result (Fig. 6B,C). In contrary, *RPL29* being the least stable reference gene when used as normalizer showed significant variation in the results (Fig. 6).

## Discussion

Most of the qRT-PCR studies in *L. erysimi*, have used reference genes from heterologous systems without prior validation in this species^5^,^6^,^7^,^8^,^9^. This becomes a serious impediment in accurate data interpretation. Such concern necessitates a comprehensive validation of the reference genes in *L. erysimi* under varied experimental conditions. In this study, 11 housekeeping genes frequently used as reference genes in other sap-sucking insects were analysed for their expression-stability in this insect-pests across the various treatment conditions^12^,^16^,^23^,^24^,^27^,^30^. The treatments *per se* as well as different intensity of treatments in terms of either dose or duration were set as the variables for assessing expression-stability of the reference genes. Perception of the treatments in the aphid samples was validated by anticipated change in marker gene-expression. In pea aphid, expression of *AP-1*gene is regulated by metamorphotic transition and increases gradually as the individual nymphs mature to alate (winged) adults^34^. Therefore, differential expression of *AP-1* validated true stage-difference of the aphids from different developmental stages (Fig. 2A). In specialist aphids like *L. eysimi* and *Brevicoryne brassicae,* myrosinase sequesters plant glucosinolates into crystalline microbodies and its expression is activated by glucosinolates in a dose dependent manner^35^,^36^,^37^. A commensurate increase in myrosinase expression in *L. erysimi* with increasing dose and duration of sinigrin treatment confirmed true treatment-effect on the test samples (Fig. 2B,C). High temperature rapidly induces the heat shock protein *Hsp83* in the larvae and pupa of *Aedes aegypti, Tribolium castaneum* and cotton aphid *Aphis gossypii*^12^,^38^,^39^. Therefore, perception of high temperature stress at the gene-expression level was verified by transcriptional activation of *Hsp83* in the treated samples (Fig. 2D). Aphid feeds in continuous fashion and secretes honeydews which are rich in free amino acids and can be quantitatively assayed by ninhydrin dyes. The volume of secreted honeydew depends on the feeding efficiency. In bioassay, deterrence in aphid-feeding by potential insecticidal compounds has been quantitatively measured by decrease in honeydew deposition^40^,^41^,^42^. In our experiments, prolonged aphid feeding on artificial diet was monitored by continuous increase in honeydew deposition in a time course manner (Fig. 2E). Similarly, significant decline in honeydew secretion was an appropriate starvation response of the aphids (Fig. 2F). Validation of treatment-effect on the gene-expression of the samples was imperative because escapes of the treatment, if any, were likely to erroneously demonstrate null-effect of the treatment on the reference gene in the statistical analysis. Surprisingly, such authentication of treatment-effects on the test-samples has not been considered in any of the earlier studies on expression of the reference genes in multiple treatments^12^,^16^,^23^,^24^,^27^,^30^. Our study for the first time explicitly highlights the importance of validating the treatment-effects in the test-samples.

Interestingly, the raw gene-expression data clearly demonstrated profound effect of all the treatments on the reference genes; though, all of them were not affected by each treatment. Collectively, the results re-ascertained the earlier reports that no single reference gene is consistent across all the treatments^22^,^23^,^43^. The most suitable reference gene(s) for each treatment was indicated by rank order of the reference genes within the treatment. Though, rank order of the reference genes was not identical in the output of the four independent statistical analysis, an empirical consensus clearly indicated the top order reference genes for each treatment conditions. For instance, *GAPDH* was ranked most stable reference gene by geNorm as well as BestKeeper for studying gene expression regulated by developmental stages; where as NormFinder and deltaCt method ranked *EF1A* as the best. The subtle differences in ranking among the top order reference genes could be attributed to difference in algorithms of the employed softwares and sensitivities towards the co-regulated reference genes. Similar variability in stability-ranking was also evident among the probable reference genes identified in other insect species^13^,^30^,^44^,^45^. Analysis of gene-expression data across the treatments and using all the four statistical methods identified *16S, RPL13* and *RPS18* as the most stable reference genes based on their consistent top order ranking (Tables S4–S6).

There has been a long debate about the optimal number of reference genes required for qRT-PCR analysis^11^,^24^. For avoiding errors in normalization, researchers have demonstrated use of multiple reference genes instead of one^28^,^46^,^47^. In our analysis geNorm pair-wise values advocate use of two reference genes for attaining better accuracy in normalization for most of the experimental conditions such as, high temperature, antibiosis and artificial diet. However, conditions like change in developmental stage and starvation would require normalization by three or more reference genes, respectively^16^,^24^. Thus, we suggest the use of more than one reference gene in combinations for studying gene-expression by qRT-PCR in *L. erysimi*, under different experimental conditions.

In conclusion, keeping in view the economic importance of *L. erysimi* as agricultural pest, functional genomics and gene-expression study would continue to constitute an important part of basic research in this insect-pest. Hence, establishing a standardized qRT-PCR in *L. erysimi* will benefit the peers in this research area. Our study empirically demonstrates that the commonly known reference genes may not be suitable for normalization in qRT-PCR without prior validation across the regime of the experimental treatments. Nevertheless, we recommend the use of *16S, RPL13*, and *RPS18*, preferably in combination for better normalization in qRT-PCR for most of the treatment conditions. This study would be a way forward to the qRT-PCR study in *L. erysimi* that are undertaken in diverse research projects worldwide owing to the growing economic importance of this insect-pest.

## Materials and Methods

### Insect rearing

Apterae adults of mustard aphids (*L. erysimi*) were collected from mustard (*B. juncea*) plants grown in the field at Indian Agricultural Research Institute, New Delhi, India during the mustard growing season, January 2015. The adults were reared on one month old mustard plants and allowed to breed repeatedly producing large number of nymphal colonies, in a growth chamber under 16 h light (140 μmol m^−2^ s^−1^), 8 h dark cycles at 22 ± 2 °C, and 62–72% relative humidity.

### Experimental treatments

#### Aphid developmental stages

Aphids of four different developmental stages, first instar nymphs, third instar nymphs, wingless and winged adults were collected from the plants grown either in the growth chamber or in the field. Twenty female aphids were released on *B. juncea* plants and allowed to lay offspring. After 24 h the first instar nymphs and after five days the third instar nymphs were collected at least from three different clonal progeny. The wingless and winged adults were collected in between 7–12 days of aphid colonies. At least 50 aphids constituting one biological replicate for each sample were flash frozen in liquid nitrogen and stored at −80 °C until used. The samples were collected for at least three biological replicates.

#### Temperature

The wingless adults were subjected to three different temperature regime, 10 °C, 22 °C and 37 °C (BOD incubator, Kuhner shaker LT-X, Switzerland) for 1 hour in Petri-dishes and harvested.

#### Starvation

For creating starvation stress, a group of 20 adult aphids were starved for 12 h and 24 h in Petri-dishes at 22 ± 2 °C in a BOD incubator. Ninhydrin test of the aphid honeydews was performed as described by Kanrar *et al*..

#### Feeding on artificial diet

A group of 20 wingless adults were maintained on artificial diet at 22 ± 2 °C in BOD incubator and harvested at different time intervals ranging from 24, 48, and 72 h.

#### Glucosinolate treatment

For the treatment of glucosinolates sinigrin at 0, 0.5, 1, 2 mg/L was supplemented in the artificial diet and a group of wingless adults were released. The aphids were collected in a time course manner at 24, 48, 72 h.

### Marker genes and biochemical test for validating treatment effects

For assessing treatment-effects on the gene expression of the treated samples either expression of a marker gene explicitly responsive to the treatment was monitored or biochemical test was performed on the samples. For aphid developmental stages, temperature and sinigrin treatment the expression of *Apterous-1* (*AP-1*), *heat shock protein (Hsp83)* and *myrosinase* (*MYR*), respectively was evaluated^12^,^34^,^35^,^36^,^37^. For treatment of feeding on artificial diet and starvation, ninhydrin assay of the honeydews was performed^48^.

### Candidate reference genes and primer design

The candidate reference genes, chosen for the present study is shown in Table S1. Primers were designed (http://www.idtdna.com/scitools/applications/primerquest/) based on their sequence in *Acyrthosiphon pisum.* Homologous sequences from *L. erysimi* were PCR amplified from cDNA and sequenced. Low quality sequence ends were trimmed by using NCBI Vecscreen (http://www.ncbi.nlm.nih.gov/tools/vecscreen/). The sequences were validated for homology using BlastN programme (Table S2). For all the qRT-PCR studies in *L. erysimi* specific primers were designed based on the obtained sequences (Table S3).

### RNA isolation and cDNA synthesis

Total RNA was isolated by using RNAiso Plus reagent (Takara Bio Inc. Tokyo, Japan) according to manufacturer’s protocol. The residual DNA was removed by using TURBO DNA free kit (Ambion Inc., UK). Yield and purity of RNA was determined by NanoDrop ND-1000 spectrophotometer (Nanodrop technologies, USA). RNA samples with an absorbance ratio OD 260/280 between 1.9–2.2 and OD 260/230 greater than 2.0, were used for further analysis. RNA-integrity was verified by resolving the samples on 1.8% agarose gel in 1X TAE. First stand cDNA was synthesized from 2 μg of total RNA in a 20-μl reaction volume using Superscript III First-Strand cDNA Synthesis Kit (Invitrogen, USA) as per manufacturer’s instruction. cDNA was stored at –20 °C for further use. For qRT-PCR analysis, each cDNA sample was diluted 20 times with nuclease free water.

### Quantitative reverse transcription-polymerase chain reaction (qRT-PCR)

All the qRT-PCR reactions were performed using SYBR green detection chemistry, in a StepOne plus real time PCR machine (Applied Biosystems, USA). A reaction cocktail of 20 μl was constituted of 2 μl diluted cDNA, 10 μl 2X SYBR Premix Ex Taq II (Takara Bio Inc, Japan), 0.4 μl of ROX reference dye and 0.4 μl each of the forward and reverse primers. PCR cycling was carried out at an initial denaturation for 30 sec at 95 °C, followed by 40 repeated cycles, each consisting of 95 °C for 10 s, 60 °C for 30 s and 72 °C for 30 s. A serial 10- fold dilution (1–1000) of cDNA was used to generate standard curve for determining the gene specific PCR efficiency (E) of the primer pairs in qRT-PCR by using linear regression model. The amplification efficiency (E) for each gene specific primers was calculated according to the equation: E (%) = (10^−*1/slope*^−1) × 100%.

### Data mining and statistical analysis

The stability of the candidate reference genes was ranked by using Microsoft Excel based software tools, geNorm^11^, NormFinder^31^, BestKeeper^32^, and deltaCt method^33^. BestKeeper uses raw data (Ct values) and PCR efficiency (E) to compute best-suited standards and combines them into an index. The geNorm program calculates an expression stability value (M) and ranks the genes in an order for given set of samples. The lower the M value, the higher is the expression stability and the M value less than 1.5 is recommended to identify stably expressed gene. It also compares the pair wise variation (V) of these genes with others. The pair wise variation value of V_n_/V_n+1_ between two sequential normalization factors was used to determine the optimal number of reference genes required for better normalization. A threshold value below 0.15 suggests the requirement of no additional reference gene for normalization^11^. NormFinder calculates gene expression stability for all the samples in any number of groups based on intra- and inter-group variations and combines these values to provide gene rank order depending on the variation in gene expression. Genes with the lowest rank are considered to be most stably expressed and are ideal to select as reference gene(s) for that particular experimental conditions. In Delta Ct method, rank order is determined based on pair-wise comparisons of gene-sets using mean delta Ct values within a particular treatment. Therefore, the average standard deviation of each gene-set is inversely proportional to the gene-expression stability.

## Additional Information

**How to cite this article**: Koramutla, M. K. *et al*. Comprehensive evaluation of candidate reference genes for qRT-PCR studies of gene expression in mustard aphid, *Lipaphis erysimi* (Kalt.). *Sci. Rep.* **6**, 25883; doi: 10.1038/srep25883 (2016).

## Supplementary Material

Supplementary Information
