# Supplementary material for: Comprehensive evaluation of candidate reference genes for qRT-PCR studies of gene expression in mustard aphid, Lipaphis erysimi (Kalt)
Source: Sci Rep. 2016 May 11;6:25883. doi: 10.1038/srep25883 (PMC4863174; doi:10.1038/srep25883)
Supplement: Supplementary Information [file srep25883-s1.doc]

**Supplementary Information**

**Comprehensive evaluation of candidate reference genes for qRT-PCR studies of gene expression in mustard aphid, *Lipaphis erysimi* (Kalt.)**

Murali Krishna Koramutla^+^, Raghavendra Aminedi ^+^, and Ramcharan Bhattacharya^*^

^1^National Research Centre on Plant Biotechnology, Indian Agricultural Research Institute Campus, New Delhi-110012, India

**Table S1. Primers from *A. pisum* used for amplifying 11 reference genes in *L. erysimi***.

| **S No** | **Gene** | **Accession No.**  **(*A. pisum*)** | **Primer sequence (5’-3’)** | **Tm**  **(ºC)** | **Product length (bp)** |
| --- | --- | --- | --- | --- | --- |
| 1 | *Succinate dehydrogenase B (SDHB)* | NM_001162436 | F: CAATACCCAACTCGACTCCTAAG  R: CTCTGAGTTCCACCAGTATGAAG | 62 | 743 |
| 2 | *16S ribosomal RNA*  *(16S)* | FJ411411 | F: TAGAAACCAACCTGGCTTACAC  R: TGACTGTGCAAAGGTAGCATAA | 62 | 485 |
| 3 | *Actin*  *(ACT)* | NM_001126200 | F: CATGGTCGGTATGGGACAAA  R:GGTAGACAGAGAAGCCAAGATG | 62 | 925 |
| 4 | *Ribosomal protein L27 (RPL27)* | NM_001126221.2 | F:CGTGCTGGTGTTATCTGGTAG  R: CGGGTCTGGAAACGGTATTT | 62 | 312 |
| 5 | *(Ribosomal protein L29) RPL29* | XM_001943721.3 | F:ACACGAGTGACAGTGGATTAAG  R:GCTGCAGCTTTCTTCTTTCTG | 62 | 363 |
| 6 | *Ribosomal protein L13 (RPL13)* | XM_001949594.3 | F:GCACCCAAGCATGGTTTATTC  R:CTACACGACGTGCTTCATAGTC | 62 | 496 |
| 7 | *Ribosomal protein s18(RPS18)* | NM_001126217.2 | F:CGTATCCTCAGCACCAACAT  R:TCCTCTACGTCCGGTAGTTT | 62 | 390 |
| 8 | *Elongation factor 1-alpha (EF1A)* | XM_008184147.1 | F:CTGATTGTGCCGTGCTTATTG  R:TGGGTGGGTTGTTCTTTGT | 62 | 678 |
| 9 | *Tubulin (TUB)* | NM_001190398 | F:GGAGCCAAGTTCTGGGAAATTA  R:CGAGATGTAAGAGGAGCGAAAC | 62 | 779 |
| 10 | *Glyceraldehyde-3-phosphate dehydrogenase (GAPDH)* | NM_001293474.1 | F:GGGATCTACTGGTGCTGATTAC  R:ACACGGTTGGAGTAACCATATT | 62 | 711 |
| 11 | *Arginine kinase (AK)* | XM_008187305.1 | F:CCATGATTCGGGAGTTGGTATT  R: CGCCTTCTGCATCTGTATGT | 62 | 782 |

**The sequence information of PCR amplified candidate reference genes from *L. erysimi* cDNA.**

> *Lipaphis erysimi* Succinate dehdrogenase B (Sdhb) mRNA

ATGGTTTTGGATGTATTGATCAAAATCAAGAACTCTGAAGATTCAACATTGACTTTTAGACGTTCCTGTCCTGTAGGTATTTGTGGTTCGTGTGCTATGAACATTGGGGGAGTCAACACATTACCCTGTCTTATTGTTGTCAATGCTGAGTTGTCTAAGCCATTGCATATATATCCTTTACCTCACATGTATGTTGTCTAG

> *Lipaphis erysimi* 16S large subunit ribosomal RNA gene rRNA GGGGTTATCTGTAGATTTGAGGTCGAACAGACCTAATACTTAAAATTTTGCACCTAAGATTAATCTTAATTCAACATCGAGGTCGCAAACTAATTTTTAAATTTGAACTTAAAAAATTAATTACGCTGTTATCCCTAAAGTAACTTTTTCCTTTAATTAAAAATTTTAATTCAAATATTCATTAAATAATGTAAAATTTTAAAAAAAGTTATTTAAATTTTTTTATCACCCCAATAAAATAAATATTAAAATTAAAATATTTATTTTCCAAAAAAATTAAAATTAATATTTATAAAGTTTTATAGGGTCTTATCGTCCCTTTAAATAATTTAAGCTTTTTAACTTAAAAATAAAATTCTAATTATATAAATAATAAAGTCTATTTCTCATCAAATCTTTCATACAAGTCCTCAATTAAAAGACTAATTATTATGCACTGGGGAAAGAGACAAAACCGAGTCGTCCATGACATTAGGAGACATTGTCTCTCCCGCTCTTTGGACTTCAAACAGGACATGACAACAACTTCCACTTCCAACGCAGTGGGATAAATCCAACTAAGTACCTGACGGACAGGTCACCCACCATCGAGAAACAGAAGTTTCCCATTTCCCCATAAACCGAATTCCAACGGTCAGTCTTAGAGAATGGTATCTTGCGGTGTCCTCGATACTGCATCCTACTCCATCAGAAATACCACG

> *Lipaphis erysimi* actin (act) mRNA

ATGGAAAAAATCTGGCATCACACTTTCTACAACGAGTTGCGTGTTGCCCCAGAAGAACACCCAATCTTGTTGACCGAAGCCCCATTGAACCCAAAGGCTAACCGTGAAAAGATGACTCAAATCATGTTTGAAACCTTCAACACACCCGCCATGTATGTTGCCATCCAAGCCGTACTCTCCTTGTACGCTTCCGGTCGTACCACTGGTATCGTTTTGGACTCTGGTGACGGTGTCTCCCACACAGTCCCCATCTATGAAGGTTATGCATTGCCCCATGCCATCCTCCGTTTGGACTTGGCTGGTCGTGACTTGACCGACTACTTGATGAAGATCTTAACCGAGAGAGGTTACAGCTTCACCACCACCGCTGAGCGAGAAATCGTCCGTGACATTAAGGAAAAATTGTGCTATGTCGCTTTGGACTTCGAACAGGAAATGGCTACCGCTGCCGCTTCCACCTCATTGGAGAAATCCTACGAATTACCTGACGGACAGGTCATCACCATCGGAAACGAACGTTTCCGTTGCCCAGAAGCCTTGTTCCAACCTTCATTCTTGGGAATGGAATCTTGCGGTATCCACGAAACTGTATACACTCCATCATGA

> *Lipaphis erysimi* ribosomal protein L27 (Rpl27) mRNA

ATGTCACAGGAAAAACTAAAAAAACGATCTAAAATCAAACCTTTCCTGAAAGTGCTCAATTACAACCACCTTATGCCAACTAGATACTCCGTTCAAGATGTAACAGTAGACAAGGTTTCACCTAAAGATTTAAAAGATCCAATGATTAAAAAGAAATACCGTCGACAGACCCGAACTGATATCCTCATGACCGCTGGGGGACGGTGTTTCCCACATACCACCCATCTACCAGCAGGTAGGCAATGCCCCGTGCCATCCTACACTTTGGACTTGGCTGGTGGTGACTTTATCGACTACTTCGCTGAACATCTTTACACGCTAGAGGTTACATTTTTTTTTTTTTTCCGCAAAGCCAGAAAACGCCCCTGA

> *Lipaphis erysimi* 60S ribosomal protein L29 (Rpl29) mRNA

ATGGCCAAGTCGAAGAATCATACCAATCACAATCAAAATCGTAAGGACCATCGTAATGGTATTTACAGGCCAAAGAAATACAGACATGAATCGCGTCGTGGTGTTTGTCAAAAGTTTTTAAGGAACCAGAAACATGCTTTAAAAGGAAATTTGTCCACTGCAGATCAAGCTGCCAGGGCTATCGAGAGGAGCGAAAAGAGGACAGCTTTGAGGACCAAGTTTGCTGAATTCAGAAAGAAGAAAGCCTGCAGCCATTTTGGTTCGCTTTAG

> *Lipaphis erysimi* 60S ribosomal protein L13a (Rpl13) mRNA ATGATCCCTCATAAAACAACTCGTGGGGCACAAGCTTTAAGACGTATGAAGGCATATGAAGATATCCCAGCTCGTTATACATCTCAAAAAACAATGGTAGTACCTTTGGCTATGAGAATGTTGAGTCTGCAACGTGGACGTAAATACTGCGATGTTGGTCGTTTATCACATGAAGTTGGATGGAAATACCAACATGTAGTCAAAGACATACGGGGTGCGAATTGCGCGCAAGTGAGACCCCCCCCCCCCCCCAGGAAAGGGAAGGATGTTCGAGACCAGGTTTTTGGGAGTTTTTGGACTTAG

> *Lipaphis erysimi* ribosomal protein S18 (Rps18) mRNA ATGTTGGAAAACCGTCTGCCGATTCCCTTGATGGCGGTCATGGCGAACATGACTTTACGCTTGCCATCGATGTTGTTGAAAGAAATACGAAAGTCAAGTGGACGTACCTACTGCGATGTTGGTATGTTTATCAGAAGAAGTTGGATGGAAATACCAACATGTAGTCAAAGACATACGGATCTTTTGGAAAAAACCTGGGTTTGGGGGGGGGGGAACTCCCCCACCACCCCCCCCGGAAAGGCAAGTCGCTCGACATGCACCACCACTCTGCGTTTCGAAGCGGGTTCGTAA

> *Lipaphis erysimi* elongation factor 1-alpha mRNA ATGGACTCAACTGAACCACCATACAGCGAAAGCCGTTTTGAAGAAATCAAGAAAGAAGTCAGCAGTTACATCAAGAAAATTGGTTACAACCCAGCTGCGGTTGCTTTTGTGCCAATCTCTGGATGGAATGGAGACAACATGTTGGAAGTTTCCGAAAAGATGTCTTGGTTCAAAGGATGGACTGTTGAACGTAAAGAAGGAAAGGCCGACGGTAAATGTTTGATTGAAGCTTTAGACGCCATCCTACCACCCAGTCGCCCAACTGACAAGGCTCTCCGTCTCCCACTCCAGGATGTGTACAAGATTGGAGGTATTGGAACAGTCCCAGTAGGTCGTGTGGAAACTGGTCTTTTAAAACCAGGTATGGTTGTGGTTTTCGCACCTGCAAACATCACCACTGAAGTCAAGTCTGTGGAGATGCACCACGAAGCTTTGGTAGAAGCTGTTCCCGGAGACAATGTTGGTTTCAATGTAAAGAACGTTTCAGTCAAAGAATTGAGACGTGGTTTCGTTGCTGGAGACACAAAGAACCCCACCCCCCACAAAAAGAAGGGGGGGGTTGAAAGAGTCATGTCCGGAACTTAA

> *Lipaphis erysimi* tubulin beta-1 (Tub)mRNA TTGGAACGTATCAATGTATACTACAATGAAGCATCAGGTGGAAAGTATGTACCACGTGCCATTTTGGTTGATTTGGAACCTGGTACCATGGACTCTGTCAGATCTGGACCATTCGGTCAAATTTTCAGACCAGACAACTTTGTCTTTGGACAATCAGGTGCTGGAAACAATTGGGCTAAAGGTCATTACACAGAGGGTGCTGAGCTTGTAGATTCAGTATTAGATGTTGTCAGGAAAGAAGCTGAGAGCTGTGATTGCCTCCAAGGTTTCCAATTGACACATTCCTTAGGTGGTGGTACTGGTTCTGGTATGGGAACCTTATTGATCTCCAAAATCCGTGAAGAATACCCAGACAGAATTATGAACACATATTCTGTTGTACCCTCTCCAAAAGTATCCGACACTGTTGTAGAACCTTACAATGCTACCCTTTCAGTTCATCAATTGGTTGAAAATACTGATGAAACCTATTGTATTGACAATGAAGCTTTGTACGACATTTGCTTCCGTACATTAAAACTTACAACACCAACATATGGTGACTTAAACCACTTGGTCTCTTTGACCATGTCTGGAGTGACCACTTGTCTAAAGTTCCCTGGTCAATTGAACGCTGATCTCCGTAAACTCGCTGTCAACATGGTTCCTTTCCCCACGTTACATTTCTTCATGCCTGGTTTCGTT

> *Lipaphis erysimi* glyceraldehydes 3-phosphate dehydrogenase (GAPDH)mRNA TTGGAAGGTGGAGCCAAGAAAGTTATCATCTCTGCACCAAGTGCTGATGCACCAATGTTTGTTGTTGGTGTTAATTTGGATGCATACAATCCATCATTCAAAGTTGTATCTAATGCTTCATGCACAACTAACTGCTTGGCTCCATTAGCCAAGGTTATTCATGATAACTTTGGAATTGTTGAAGGTCTTATGACAACTGTTCATGCTACCACCGCCACCCAAAAAACTGTTGATGGACCATCTGGAAAATTATGGAGAGATGGCAGAGGTGCTGCCCAAAACATCATTCCTGCATCTACTGGAGCAGCCAAGGCTGTAAGTAAGGTCATCCCAGAACTTAATGGAAAATTAACTGGAATGGCTTTCAGAGTACCAGTTGCTAATGTTTCTGTTGTTGACTTGACTGTAAGACTTGCAAAACCTGCTTCTTACCAAGACATCAAAAATAAAGTCAAGGAAGCTGCAGAAGGACCATTGAAAGGAATTTTGGGTTACACTGAAGATGAAGTTGTGTCTTCTGACTTTATTGGTGATACCCACTCATCAATCTTTGATGCCCAGGCGGGAATTTCATTGAACGATCAATTTGTTAAACTTATCTCATGGTACGACAATGAATATGGTATTAC

> *Lipaphis erysimi* arginine kinase-like (AK) mRNA CTGGATCCAGAATGCAAGTATATAATTTCTACAAGAGTACGTTGTGGCCGTTCACTGGAAGGATATCCGTTCAATCCATTGTTGACCGAACATCAGTACAAGGAGATAGAGAATAAGGTTTCTTGCACGCTCACAGGACTTAGCGGCGAATTGAAAGGAGAGTTTTATCCCTTAACTGGCATGTCTAAAGATGTACAGCAGAAATTAATCGATGATCATTTTCTATTTAAGGAAGGTGATAGATTTTTACAGGCTGCTAACGCTTCGCGATTCTGGCCAACCGGCCGTGGTATATTCCATAACGAAAACAAAACGTTCCTGGTTTGGTGTAATGAGGAAGATCATTTAAGGCTCATATCCATGCAAAAAGGAGGAAACTTGGGAGAGGTATATGCAAGACTCGTAACAGCGGTAAACGATGTGGAAAAAAGACTTCCTTTTTCGCATCACAACCGGTTGGGATTTTTAACATTCTGTCCAACAAACTTGGGGACAACTATCCGTGCATCAGTTCATATTCAGCTGCCTAAATTAGCTGCAGACAGGCCAAATTGGAAGAAATTGCTGGAAAATATAATTTACAAGTCAGAGGTACTCGTGGAGAACATACGGCC

| Gene | Primer sequence (5’-3’) | Product length (bp) | Tm  (^0^ C) | Primer efficiency  (%E) | Regression  coefficient (R^2^) |
| --- | --- | --- | --- | --- | --- |
| *SDHB* | F:CTGAGTTCTTGTGGACCTATG  R:CCACAAATACCTACAGGACAG | 104 | 62 | 98.1 | 0.9999 |
| *16S* | F:ATAGGGTCTTATCGTCCCTTTA  R:GGACTTGTATGAAAGATTTGATGAG | 109 | 62 | 92.5 | 0.9999 |
| *ACT* | F: GTGACTTGACCGACTACTTGAT  R: TCGAAGTCCAAAGCGACATAG | 124 | 62 | 102.4 | 0.9931 |
| *RPL27* | F: GATCGTTACCCTCGGAAAGTC  R:CGGAGTATCTAGTTGGCATAAGG | 121 | 62 | 93.3 | 0.9979 |
| *RPL29* | F:CGCTTGTCTTCGCGTTTAAG  R:GACGCGATTCATGTCTGTATTTC | 143 | 62 | 99.0 | 0.9879 |
| *RPL13* | F:GATATCCCAGCTCGTTATACATCTC  R:ACGACCAACATCGCAGTATT | 102 | 62 | 93.1 | 0.9986 |
| *RPS18* | F: GGTACATTCTCCGGCTCTTT  R:GGCAAGCGTAAAGTCATGTTC | 117 | 62 | 95.1 | 0.9989 |
| *EF1A* | F:GAAGCTTTAGACGCCATCCTAC  R:GGACTGTTCCAATACCTCCAATC | 100 | 62 | 93.3 | 1.0000 |
| *TUB* | F:GGGTGCTGAGCTTGTAGATT  R:CAGTACCACCACCTAAGGAATG | 107 | 62 | 97.5 | 0.9999 |
| *GAPDH* | F:TGGCTTTCAGAGTACCAGTTG  R:TCTGCAGCTTCCTTGACTTTAT | 109 | 62 | 91.2 | 1.0000 |
| *AK* | F:CGTTCCTGGTTTGGTGTAATG  R:TACCGCTGTTACGAGTCTTG | 101 | 62 | 101.4 | 0.9988 |

| **S. No.** | ***L. erysimi* genes** | **BlastN** | | **Accession Number** | **Aphid Species** |
| --- | --- | --- | --- | --- | --- |
|  |  | **Identity** | ***E*-value** |  |  |
| 1 | *Succinate dehydrogenase B (SDHB)* | 84% | 0.0 | [FP925039.1](http://www.ncbi.nlm.nih.gov/nucleotide/299885798?report=genbank&log$=nucltop&blast_rank=1&RID=H4T3H86N016) | *Acyrthosiphon pisum* |
| 2 | *16S large subunit ribosomal RNA (16S)* | 100% | 0.0 | EU358891.1 | *Lipaphis pseudobrassicae* |
| 3 | *Actin (ACT)* | 97% | 0.0 | NM_001142636.1 | *Acyrthosiphon pisum* |
| 4 | *Ribosomal protein L27 (RPL27)* | 94% | 5e-111 | [NM_001126221.2](http://www.ncbi.nlm.nih.gov/nucleotide/209969791?report=genbank&log$=nuclalign&blast_rank=1&RID=H4V46YXC01R) | *Acyrthosiphon pisum* |
| 5 | *60S ribosomal protein L29 (RPL29)* | 91% | 8e-126 | [XM_001943721.3](http://www.ncbi.nlm.nih.gov/nucleotide/641660111?report=genbank&log$=nuclalign&blast_rank=1&RID=H4VR3NK4013) | *Acyrthosiphon pisum* |
| 6 | *60S ribosomal protein L13a (RPL13)* | 95% | 0.0 | [XM_001949594.3](http://www.ncbi.nlm.nih.gov/nucleotide/641653154?report=genbank&log$=nucltop&blast_rank=1&RID=H4W072HN013) | *Acyrthosiphon pisum* |
| 7 | *Ribosomal protein S18 (RPS18)* | 95% | 2e-146 | [NM_001126217.2](http://www.ncbi.nlm.nih.gov/nucleotide/209693453?report=genbank&log$=nuclalign&blast_rank=2&RID=H4W6VCRV016) | *Acyrthosiphon pisum* |
| 8 | *Elongation factor 1-alpha (EF1A)* | 94% | 0.0 | [XM_008184147.1](http://www.ncbi.nlm.nih.gov/nucleotide/641662723?report=genbank&log$=nucltop&blast_rank=1&RID=H4WKXC0X016) | *Acyrthosiphon pisum* |
| 9 | *Tubulin beta-1 (TUB)* | 96% | 0.0 | [NM_001190398.1](http://www.ncbi.nlm.nih.gov/nucleotide/298676438?report=genbank&log$=nucltop&blast_rank=1&RID=H4WR70XB013) | *Acyrthosiphon pisum* |
| 10 | *Glyceraldehyde-3-phosphate dehydrogenase (GAPDH)* | 96% | 0.0 | [NM_001293474.1](http://www.ncbi.nlm.nih.gov/nucleotide/648216013?report=genbank&log$=nucltop&blast_rank=1&RID=H4X1CD13016) | *Acyrthosiphon pisum* |
| 11 | *Arginine kinase-like (AK)* | 93% | 0.0 | [XM_008187305.1](http://www.ncbi.nlm.nih.gov/nucleotide/641671271?report=genbank&log$=nucltop&blast_rank=1&RID=H4X5JFPN01R) | *Acyrthosiphon pisum* |

**Table S2. Homology of PCR amplified candidate reference genes in *L. erysimi*.**

**Table S3. Primers used for studying reference gene expression in *L. erysimi* by qRT-PCR.**

**Table S4. Ranking of candidate reference genes calculated by BestKeeper based on their expression stability.**

| **Rank** | **Developmental stages** | | **Starvation** | | **Temperature** | | **Glucosinolate** | | **Artificial diet** | | **Total samples** | |
| --- | --- | --- | --- | --- | --- | --- | --- | --- | --- | --- | --- | --- |
|  | **gene** | **SD^a^** | **gene** | **SD** | **gene** | **SD** | **gene** | **SD** | **gene** | **SD** | **gene** | **SD** |
| 1 | *GAPDH* | 0.05 | *16S* | 0.40 | *16S* | 0.04 | *RPL13* | 0.96 | *RPL27* | 0.11 | *16S* | 0.92 |
| 2 | *16S* | 0.14 | *RPS18* | 0.69 | *RPL13* | 0.15 | *RPS18* | 1.00 | *RPL29* | 0.84 | *RPL13* | 1.00 |
| 3 | *EF1A1A* | 0.46 | *RPL13* | 0.78 | *RPL27* | 0.17 | *16S* | 1.12 | *SDHB* | 1.12 | *RPS18* | 1.17 |
| 4 | *RPS18* | 0.49 | *TUB* | 1.02 | *TUB* | 0.26 | *RPL29* | 1.12 | *RPL13* | 1.12 | *ACT* | 1.22 |
| 5 | *RPL27* | 0.53 | *ACT* | 1.62 | *EF1A* | 0.30 | *TUB* | 1.26 | *ACT* | 1.25 | *TUB* | 1.33 |
| 6 | *AK* | 0.74 | *SDHB* | 3.41 | *ACT* | 0.44 | *ACT* | 1.31 | *TUB* | 1.38 | *RPL27* | 1.87 |
| 7 | *TUB* | 0.81 | *RPL27* | 3.47 | *AK* | 0.54 | *RPL27* | 1.96 | *16S* | 1.66 | *SDBH* | 2.45 |
| 8 | *RPL13* | 0.97 | *AK* | 4.65 | *SDHB* | 0.68 | *SDHB* | 2.05 | *RPS18* | 2.33 | *AK* | 3.88 |
| 9 | *ACT* | 1.02 | *EF1A* | 6.26 | *GAPDH* | 0.85 | *AK* | 4.10 | *AK* | 3.80 | *GDH* | 5.39 |
| 10 | *SDHB* | 2.30 | *GAPDH* | 7.14 | *RPS18* | 1.23 | *EF1A* | 5.56 | *GAPDH* | 5.45 | *RPL29* | 5.40 |
| 11 | *RPL29* | 2.45 | *RPL29* | 7.93 | *RPL29* | 8.39 | *GAPDH* | 5.84 | *EF1A* | 5.65 | *EF* | 5.52 |

^a^ refers to the standard deviation

**Table S5. Ranking of candidate reference genes calculated by NormFinder based on their expression stability.**

| **Rank** | **Developmental stages** | | | **Starvation** | | **Temperature** | | **Glucosinolate** | | **Artificial diet** | | | **Total samples** | |
| --- | --- | --- | --- | --- | --- | --- | --- | --- | --- | --- | --- | --- | --- | --- |
|  | **gene** | | **SV^b^** | **gene** | **SV** | **gene** | **SV** | **gene** | **SV** | **gene** | | **SV** | **gene** | **SV** |
| 1 | *EF1a* | 0.003 | | *16S* | 0.005 | *EF1a* | 0.001 | *RPS18* | 0.020 | *RPS18* | 0.018 | | *RPS18* | 0.019 |
| 2 | *RPS18* | 0.003 | | *RPS18* | 0.005 | *RPL27* | 0.001 | *RPL27* | 0.021 | *AK* | 0.022 | | *ACT* | 0.020 |
| 3 | *16S* | 0.005 | | *SDBH* | 0.023 | *RPL13* | 0.002 | *ACT* | 0.024 | *ACT* | 0.023 | | *TUB* | 0.020 |
| 4 | *GDH* | 0.006 | | *TUB1b* | 0.029 | *SDBH* | 0.003 | *SDBH* | 0.025 | *RPL13* | 0.024 | | *RPL13* | 0.022 |
| 5 | *RPL27* | 0.008 | | *RPL13* | 0.030 | *TUB1b* | 0.004 | *RPL29* | 0.025 | *TUB1b* | 0.024 | | *AK* | 0.031 |
| 6 | *TUB1b* | 0.010 | | *AK* | 0.047 | *AK* | 0.005 | *AK* | 0.029 | *SDBH* | 0.029 | | *SDBH* | 0.032 |
| 7 | *AK* | 0.017 | | *ACT* | 0.067 | *ACT* | 0.013 | *RPL13* | 0.031 | *RPL29* | 0.031 | | *RPL27* | 0.046 |
| 8 | *RPL13* | 0.017 | | *RPL27* | 0.079 | *16S* | 0.016 | *TUB1b* | 0.031 | *RPL27* | 0.044 | | *GDH* | 0.068 |
| 9 | *ACT* | 0.021 | | *EF1a* | 0.100 | *GDH* | 0.022 | *16S* | 0.034 | *EF1a* | 0.048 | | *EF* | 0.070 |
| 10 | *SDBH* | 0.039 | | *GDH* | 0.113 | *RPS18* | 0.023 | *EF1a* | 0.068 | *GDH* | 0.061 | | *16S* | 0.073 |
| 11 | *RPL29* | 0.043 | | *RPL29* | 0.154 | *RPL29* | 0.169 | *GDH* | 0.078 | *16S* | 0.107 | | *RPL29* | 0.093 |

^b^ refers to the stability value

**Table S6. Ranking of candidate reference genes calculated by Delta Ct method based on their expression stability.**

| **Rank** | **Developmental stages** | | | **Starvation** | | **Temperature** | | **Glucosinolate** | | **Artificial diet** | | | **Total samples** | |
| --- | --- | --- | --- | --- | --- | --- | --- | --- | --- | --- | --- | --- | --- | --- |
|  | **gene** | | **SD^c^** | **gene** | **SD** | **gene** | **SD** | **gene** | **SD** | **gene** | | **SD** | **gene** | **SD** |
| 1 | *EF1a* | 1.24 | | *16S* | 4.77 | *16S* | 1.51 | *16S* | 2.08 | *ACT* | 2.42 | | *RPS18* | 3.32 |
| 2 | *RPS18* | 1.24 | | *RPS18* | 4.79 | *EF1a* | 1.53 | *SDBH* | 2.15 | *RPL13* | 2.42 | | *TUB* | 3.37 |
| 3 | *16S* | 1.29 | | *TUB1b* | 5.08 | *RPL27* | 1.57 | *RPS18* | 2.15 | *SDBH* | 2.43 | | *RPL13* | 3.42 |
| 4 | *GDH* | 1.34 | | *RPL13* | 5.24 | *RPL13* | 1.57 | *RPL29* | 2.18 | *TUB1b* | 2.46 | | *16S* | 3.52 |
| 5 | *RPL27* | 1.35 | | *ACT* | 5.69 | *ACT* | 1.58 | *ACT* | 2.24 | *16S* | 2.48 | | *SDBH* | 4.06 |
| 6 | *TUB1b* | 1.40 | | *SDBH* | 5.76 | *AK* | 1.66 | *RPL13* | 2.33 | *RPL29* | 2.58 | | *RPL27* | 4.13 |
| 7 | *RPL13* | 1.62 | | *AK* | 6.49 | *TUB1b* | 1.77 | *RPL27* | 2.59 | *RPS18* | 2.82 | | *AK* | 4.33 |
| 8 | *ACT* | 1.75 | | *RPL27* | 7.15 | *RPS18* | 1.77 | *AK* | 2.99 | *RPL27* | 3.20 | | *ACT* | 4.33 |
| 9 | *AK* | 1.84 | | *EF1a* | 7.89 | *SDBH* | 1.78 | *TUB1b* | 3.53 | *AK* | 3.88 | | *GDH* | 5.42 |
| 10 | *SDBH* | 3.23 | | *GDH* | 8.90 | *GDH* | 1.87 | *GDH* | 4.69 | *EF1a* | 5.53 | | *EF* | 5.58 |
| 11 | *RPL29* | 3.99 | | *RPL29* | 12.77 | *RPL29* | 11.52 | *EF1a* | 7.29 | *GDH* | 5.71 | | *RPL29* | 7.65 |

^c^ refers to the average standard deviation


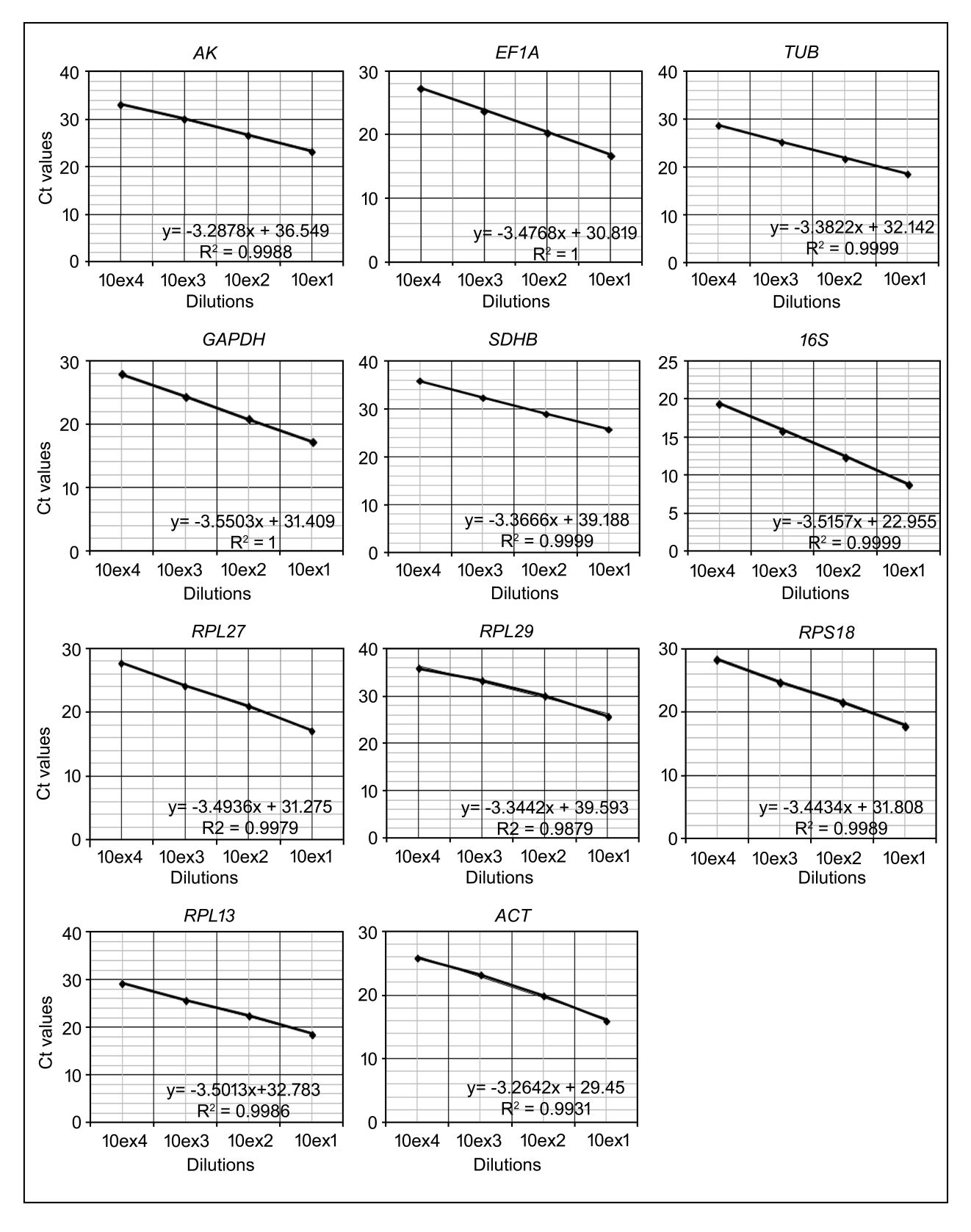
**Figure S1. Regression analysis showing amplification efficiency of the qRT-PCR primers.**
